# Factors associated with perceived quality of life in older adults: ELSI-Brazil

**DOI:** 10.11606/S1518-8787.2018052000613

**Published:** 2018-10-25

**Authors:** Anita Liberalesso Neri, Flávia Silva Arbex Borim, Arlete Portella Fontes, Dóris Firmino Rabello, Meire Cachioni, Samila Sathler Tavares Batistoni, Mônica Sanches Yassuda, Paulo Roberto Borges de Souza, Fabiola Bof de Andrade, Maria Fernanda Lima-Costa

**Affiliations:** I Universidade Estadual de Campinas. Faculdade de Ciências Médicas. Programa de Pós-Graduação em Gerontologia. Campinas, SP, Brasil; II Universidade do Recôncavo da Bahia. Centro de Ciências da Saúde. Santo Antônio de Jesus, BA, Brasil; III Universidade de São Paulo. Escola de Ciências, Artes e Humanidades. Curso de Gerontologia. São Paulo, SP, Brasil; IV Fundação Oswaldo Cruz. Instituto de Comunicação e Informação Científica e Tecnológica em Saúde.Brasil; Ministério da Saúde. Belo Horizonte, MG, Brasil; V Fundação Oswaldo Cruz. Instituto René Rachou. Programa de Pós-Graduação em Saúde Coletiva. Belo Horizonte, MG, Brasil; VI Fundação Oswaldo Cruz. Instituto René Rachou. Núcleo de Estudos em Saúde Pública e Envelhecimento. Belo Horizonte, MG, Brasil

**Keywords:** Aged, Quality of Life, Self-Assessment, Socioeconomic Factors, Community Networks, Health Surveys, Idoso, Qualidade de Vida, Autoavaliação, Fatores Socioeconômicos, Redes Comunitárias, Inquéritos Epidemiológicos

## Abstract

**OBJECTIVE:**

To identify factors associated with perceived quality of life in a representative national sample of the population aged 50 or over.

**METHODS:**

Data from 7,651 participants of the baseline ELSI-Brazil (Brazilian Longitudinal Study of Aging), conducted between 2015 and 2016, were used. The perceived quality of life was measured by the CASP-19 scale - (CASP - control, autonomy, self-fulfillment and pleasure), considering the highest tertile as good quality of life. The independent variables included socio-demographic characteristics, mobility, loneliness, and indicators of sociability (social network, social support and social participation). The associations were tested using multivariate Poisson regression.

**RESULTS:**

The best perceived quality of life showed a positive and independent association with the frequency of contacts with friends (PR = 1.25 for at least once every 2–3 months and PR = 1.36 for at least once a week), instrumental support from spouse or partner in the household (PR = 1.69), and emotional support from other relatives (PR = 1.45), children or children in law (PR = 1.41) and spouse or partner (PR = 1.33). Negative associations were observed for participants aged 80 and over (RP = 0.77), with 4 to 7 or 8 or more years of schooling (PR = 0.78 and 0.75, respectively) and with difficulty in mobility (PR = 0.83).

**CONCLUSIONS:**

In addition to age and schooling, mobility, sociability and instrumental and emotional support are associated with perceived quality of life among older Brazilian adults. These characteristics must be considered when actions are taken, aiming to promote quality of life in this population.

## RESUMO

**OBJETIVO:**

Identificar fatores associados à qualidade de vida percebida em amostra nacional representativa da população com 50 anos ou mais.

**MÉTODOS:**

Foram utilizados dados de 7.651 participantes da linha de base do ELSI-Brasil, conduzida entre 2015 e 2016. A qualidade de vida percebida foi aferida pela escala CASP-19, considerando-se como boa qualidade de vida percebida o tercil mais alto. As variáveis independentes incluíram características sociodemográficas, mobilidade, solidão, redes sociais, apoio social e participação social. As associações foram testadas por meio de regressão de Poisson multivariada.

**RESULTADOS:**

A melhor qualidade de vida percebida apresentou associação positiva e independente com a frequência de encontros com amigos (RP = 1,25 para pelo menos uma vez a cada 2–3 meses e RP = 1,36 para pelo menos uma vez por semana), suporte instrumental dentro de casa, de cônjuge ou companheiro (RP = 1,69) e suporte emocional de outros parentes (RP = 1,45), de filho, nora ou genro (RP = 1,41) e do cônjuge ou companheiro(a) (RP = 1,33). Associações negativas foram observadas para idade igual ou superior a 80 anos (RP = 0,77), escolaridade igual a 4–7 e 8 anos ou mais (RP = 0,78 e 0,75, respectivamente) e dificuldade na mobilidade (RP = 0,83).

**CONCLUSÕES:**

Além da idade e da escolaridade, a mobilidade, a sociabilidade e o suporte instrumental e emocional estão associados à qualidade de vida percebida entre adultos mais velhos brasileiros. Essas características devem ser consideradas nas ações visando à promoção da qualidade de vida dessa população.

## INTRODUCTION

The most frequently used models in research on old age include measures of health conditions, such as morbidity, frailty, mortality and disability; and socioeconomic status, such as income, schooling and housing arrangements^1^ ^-^ ^5^. This is understandable, once these measures serve to guide decision-making regarding public policies for old age, mainly when it is associated with declines in health and functionality. Even in countries with good formal support systems, family and friends, acquaintances and volunteers are considered highly relevant for the protection and care of the elderly. The structure and functionality of these informal social networks can protect both the health and well-being of the elderly from the effects of losses and adversities, as well as promote the continuity of their development^6^. The size of the social networks, the kinds of bonds and the frequency of interactions between the members, the availability of social support and the levels of social participation exhibited by the elderly are consistently associated with patterns of morbidity, mortality, and physical and cognitive functioning^6–9^.

Subjective variables have been considered as a comparable or more robust predictor of mortality, morbidity and disability than objective indicators of physical, economic and social well-being^10–15.^ For this reason, they have been increasingly integrated into the assessment of quality of life in adults and aged individuals. In addition, it is suggested that they are considered as focal points of public policies aimed at these groups^16^. They comprise conditions such as loneliness^4,10–12^; perception of sufficiency of monthly family income to meet personal and family needs^15^; judgments of the support coming from members of informal social networks ^6^ ^,^ ^17^ ^,^ ^18^; and psychological well-being, identified with the eudaimonic definition of happiness as a search for personal excellence e and a sense of worth, purpose, and personal adjustment ^19^ ^,^ ^20^.

The operationalization of the concept of quality of life in eudaimonic terms is performed by the CASP-19, a scale designated by the acronym formed by the initials of the names of its factors: *control*, *autonomy*, *self-realization and pleasure* ^1^ ^,^ ^21^. For Hyde et al.^21^, the measure of quality of life must be distinguished from the contextual and individual phenomena capable of influencing it, such as health, social networks and material circumstances. The CASP-19 refers to the Maslow model of basic needs, seen as ontological or inherent to human nature. Control is defined as the ability to actively intervene in the environment, and autonomy is understood as the right of an individual to be free from undesirable interference on the part of others^1^ ^,^ ^21^. Self-realization and pleasure reflect the way by which an adult or aged individual perceive him or herself as active agent of his or her own life^1^ ^,^ ^21^.

As far as we know, there are no Brazilian studies with national coverage regarding the quality of life of older adults. Additionally, there are no populational studies that have adopted a psychological approach to perceived quality of life (PQOL) using the CASP-19. The objective of this study was to describe the variations in the quality of life in a representative national sample of older adults, considering socio-demographic characteristics, mobility and sociability indicators.

## METHODS

### Data Source

For this analysis, there were data from the baseline of the Brazilian Longitudinal Study of Aging (ELSI-Brazil) conducted between 2015 and 2016. The sample consisted of 8,424 older adults selected from the 9,412 participants of the baseline ELSI-Brazil, who responded to the items of interest for this study (psychosocial module) without the help of a proxy. The sample was designed to represent the non-institutionalized Brazilian population aged 50 and over, residing in 70 cities located in the five geographic regions of the country. The ELSI-Brazil sampling used a design with selection stages combining stratification of primary sampling units (cities), census tracts, and households. These cities were allocated into four strata, depending on their population size. All residents aged 50 years or more of the selected households were eligible for individual interview. Detailed information on sampling, the variables and the instruments, and ELSI-Brazil data collection are found in the electronic research site^a^ and in the previous publication by Lima-Costa et al.^22^

### Variables

The dependent variable was perceived quality of life (PQOL), evaluated using the CASP-19, a scale with 19 Likert–type items (from never = 0 to always = 3) which refers to the domains of control, autonomy, self-realization and pleasure, whose acronym derives from its name. The total score ranges from zero to 57. Participants are invited to evaluate to what extent each item describes their feelings about their own life ^1^ ^,^ ^21^ ^,^ ^23^. Those who scored in the highest tertile (48 points or more) were considered to have the best PQOL.

The independent variables of the present study included the following: socio-demographic characteristics (age, sex and schooling), mobility, feeling of loneliness, social network, social support and social participation. To evaluate mobility, four items were used: the degree of difficulty to walk 100 meters, to climb a flight of stairs, to climb several flights of stairs without stopping or resting, and to walk 1 km continuously. The difficulty in mobility was attributed to those who reported that they had some or significant difficulty to carry out one or more of the abovementioned activities^16^. Loneliness was defined by the emotional experience of being alone, socially isolated, or deprived of expected or significant relationships or interactions^10–12^. Loneliness was evaluated through the following item of the Center for Epidemiological Scale – Depression (CES-D)^24^: “How often do you feel alone or lonely?” (never, sometimes or always). Social interactions were measured by the frequency of face-to-face interactions with children, other relatives and friends in the last 12 months ^16^.

The levels of social participation were indicated by the involvement in eight types of social activities selected of an inventory containing 14 advanced activities of daily living (AADL), defined as social and leisure activities of a discretionary nature, which mirror preferences, motivations and individual abilities, influenced by psychosocial factors^25^. These activities were categorized according to the requirements of the environment (from small, imposed by more restricted ones, to large, imposed by the ones that are more open) and according to the degree of complexity of activities (with a lower or higher requirement for independence and autonomy)^26^. At the proximal level, there were the following AADL: (1) to invite others to go to your own home; (2) to visit friends or family members in their respective homes; and (3) to maintain contact with others through letters, telephone, email, social networks and the Internet; at the intermediate level there were: (4) to meet with friends to play table games; and (5) to hang out with other people to go to public places, such as restaurants, clubs, and squares; and at the distal level there were: (6) to participate in organized social activities or community groups; (7) to perform voluntary work; and (8) to participate in civil associations, political parties, councils or boards. The items were dichotomous and referred to the previous 12 months. Positive responses were added at each level. In this analysis, the participations at the proximal, intermediate and distal levels were categorized as “yes” (has at least one activity) and “no”.

The sources of support were determined by participants’ expectations about who, if necessary, would offer them help to care for their home, to carry out tasks outside the home, to be their confidant and to lend them money or objects. The answers were categorized into: spouse or partner, children or daughter-in-law or son-in-law, other relatives, others (friends, maid, paid caregiver, neighbors) and no one.

### Statistical Analysis

The analyses of associations of the independent variables with the dependent variable were performed using prevalence ratios and their confidence intervals (95%CI) were estimated with Poisson regression. Initially, the association between each independent variable and the PQOL (a dichotomous variable categorized as upper tertile *versus* lower tertile), adjusted for age and sex, was analyzed. Next, the final multiple regression model was developed. The final model was simultaneously adjusted by all independent variables, since no collinearity between them was identified (average of the variance inflation factor = 1.22). Data analysis was performed with the *svy* commands of the Stata software, version 14.0 (Stata Corporation, College Station, United States), which incorporates the weights originated from the sampling design.

### Ethical Aspects

The ELSI-Brazil was approved by the Research Ethics Committee of the Oswaldo Cruz Foundation, Minas Gerais, Brazil (CAAE - Presentation of the Certificate for Ethical Appreciation: 34649814.3.0000.5091). All participants signed an informed consent form to participate in the study.

## RESULTS

Among the 8,424 participants who responded to the psychosocial module without the help of a proxy, 7,651 provided complete information for all variables, which is why they were included in the analysis. Of these, 2,227 were classified as having good PQOL. Among participants, the mean age was 61.0 years, 53.2% were women and 38.3% had less than four years of schooling. The other characteristics of sample participants are shown in Table 1. Table 2 shows the results of the analysis adjusted for age and sex of the association between PQOL and socio-demographic characteristics, mobility and feeling of loneliness. Men, compared with women, had a better PQOL evaluation. The worst evaluation was observed for those aged equal to or older than 80 years, and for participants with 4 to 7 or 8 or more years of schooling.

**Table 1:** Characteristics of the 7,651 sample participants. Brazilian Longitudinal Study of Aging (ELSI-Brazil), 2015-2016.

Characteristic	Percent or average	95%CI
Female sex	46.8	43.6–49.4
Average age	61.9	61.2–62.6
Schooling < 4 years	38.3	35.5–41.2
Difficulty in mobility	53.8	51.1–56.5
Always feels lonely	14.9	13.5–16.5
Has no children or meets with them < 1 time per year	29.0	27.0–31.1
Has no other relatives or meets them < 1 time per year	17.2	15.7–18.9
Has no friends or meets them < 1 time per year	12.0	10.7–13.4
Participates in social activities: proximal level	89.6	87.4–91.4
Participates in social activities: intermediate level	52.1	48.4–55.8
Participates in social activities: distal level	55.3	52.3–58.3
Main instrumental support in the home: children/ daughter-in-law or son-in-law	42.8	40.8–44.8
Main instrumental support outside the home: children/ daughter-in-law or son-in-law	49.7	47.6–51.8
Primary emotional support: children/ daughter-in-law or son-in-law	34.3	32.2–36.5
Main material support: children/ daughter-in-law or son-in-law	35.1	33.1–37.1

All data are expressed as percentages, except when specified.

All estimates consider the weight of the individuals and the sample parameters.

**Table 2:** Prevalence and prevalence ratio (PR) of the best perceived quality of life (PQOL)a, according to socio-demographic variables, mobility and loneliness. Brazilian Longituginal Study of Aging (ELSI-Brazil), 2015-2016.

Variable	Prevalence of the best PQOL^a^	RP adjusted by sex and age^b^	95%CI
Gender			
Female	26.0	1	
Male	30.3	1.16	1.04–1.28^c^
Age group (years)			
50–59	28.7	1	
60–69	27.6	0.96	0.86–1.07
70–79	27.9	0.98	0.84–1.14
80 or over	23.0	0.80	0.68–0.95^d^
Schooling (years)			
< 4	32.9	1	
4–7	26.1	0.77	0.69–0.86
8 or over	25.8	0.75	0.68–0.83
Difficulty in mobility			
No	31.0	1	
Yes	25.5	0.84	0.74–0.95
Feeling of loneliness			
Never/ Sometimes	28.0	1	
Always	28.4	1.05	0.93–1.19

^a^ Defined by punctuation in the upper tertile of CASP-19.

^b^ Prevalence ratio estimated by Poisson regression.

^c^ Adjusted by age group.

^d^ Adjusted by sex.

Among social interactions, only the frequency of contacts with friends showed a statistically significant association with the best PQOL, after adjustment for age and sex. The best PQOL was also associated with participation in social activities at the distal level (Table 3). Except for the expectation of instrumental support outside the home, all the other expectations presented associations with PQOL, regardless of sex and age. They were as follows: instrumental support in the home (from other people, children, daughter-in-law or son-in-law and spouse or partner); emotional support (from other relatives, children, daughter-in-law or son-in-law and spouse or partner) and material support (from a son, daughter-in-law or son-in-law) (Table 4). Table 5 revealed the variables that presented statistically significant associations (p < 0.05) with PQOL in the final multivariate analysis. The best PQOL showed a positive and independent association with the frequency of contacts with friends (PR = 1.25 for at least once every 2–3 months and PR = 1.36 for at least once a week), instrumental support within the home from spouse or partner (PR = 1.69) and emotional support from other relatives (PR = 1.45), children, daughter-in-law or son-in-law (PR = 1.41) and the spouse or partner (PR = 1.33). Negative associations were observed for age equal to or older than 80 years (PR = 0.77), schooling equal to 4-7 and 8 years or more (PR = 0.78 and 0.75, respectively) and difficulty in mobility (PR = 0.83).

**Table 3:** Prevalence and prevalence ratio (PR) of the best perceived quality of life (PQOL)a, according to the frequency of face-to-face social interactions with family members and previous friends and social participation in the last 12 months. Brazilian Longitudinal Study of Aging (ELSI-Brazil), 2015-2016.

Variable	Prevalence of the best PQOL^a^	PR adjusted by sex and age^b^	95%CI
Meeting with children			
Has no children or meets them less than once a year	27.7	1	
1–2 times a year	31.1	1.13	0.96–1.33
At least once, every 2–3 months	29.0	1.06	0.91–1.23
1 or more times per week	27.4	1.01	0.89–1.14
Meeting with other relatives			
Has no relatives or meets them less than once a year	27.7	1	
1–2 times a year	29.4	1.05	0.93–1.19
At least once, every 2–3 months	27.2	0.97	0.85–1.10
1 or more times per week	28.1	1.00	0.90–1.11
Frequency with which one meets a friend			
Has no friends or meets them less than once a year	21.1	1	
1 or 2 times a year	22.9	1.10	0.77–1.57
At least once, every 2–3 months	26.6	1.25	1.01–1.55
1 or more times per week	29.7	1.39	1.18–1.63
Participation in social activities: proximal level			
No	27.6	1	
Yes	28.1	1.07	0.87–1.34
Participation in social activities: intermediate level			
No	28.2	1	
Yes	27.9	1.06	0.94–1.18
Participation in social activities: distal level			
No	27.0	1	
Yes	28.9	1.11	1.01–1.22

^a^ Defined by punctuation in the upper tertile of CASP-19.

^b^ Prevalence ratio estimated by Poisson regression.

**Table 4:** Prevalence and prevalence ratio (PR) of the best perceived quality of life (PQOL)a, according to the sources of instrumental, emotional and material support. Brazilian Longitudinal Study of Aging (ELSI-Brazil), 2015-2016.

Variable	Prevalence of the best PQOL^a^	PR adjusted by sex and age^b^	95%CI
Instrumental support indoors			
No one	15.9	1	
Others	26.9	1.67	1.10–2.53
Other relatives	25.2	1.54	0.99–2.39
Children/ Daughter-in-law or son-in-law	28.0	1.77	1.16–2.69
Spouse or partner	31.4	1.86	1.23–2.81
Instrumental support outside the home			
No one	19.4	1	
Others	26.0	1.34	0.86–2.09
Other relatives	27.2	1.39	0.94–2.07
Children/ Daughter-in-law or son-in-law	28.1	1.47	0.99–2.19
Spouse or partner	29.1	1.45	0.95–2.21
Emotional support			
No one	18.9	1	
Others	24.6	1.31	0.99–1.74
Other relatives	27.7	1.46	1.14–1.87
Children/ Daughter-in-law or son-in-law	29.3	1.59	1.26–2.02
Spouse or partner	30.2	1.51	1.18–1.92
Material support			
No one	24.8	1	
Others	27.7	1.09	0.89–1.34
Other relatives	26.2	1.04	0.86–1.25
Children/ Daughter-in-law or son-in-law	29.1	1.21	1.01–1.44
Spouse or partner	30.5	1.21	0.96–1.52

^a^ Defined by punctuation in the upper tertile of CASP-19.

^b^ Prevalence ratio estimated by Poisson regression.

**Table 5:** Variables that showed statistically significant associations (p < 0.05) with perceived quality of life (PQOL)* in the multivariate final analysis. Brazilian Longitudinal Study of Aging (ELSI-Brazil), 2015-2016.

Variable	adjusted PR*	95%CI
Age group (*versus* 50–59 years)		
60–69	0.92	0.83–1.03
70–79	0.91	0.78–1.08
80 or over	0.77	0.65–0.92
Schooling (*versus* < 4 years)		
4–7	0.78	0.69–0.88
8 or over	0.75	0.66–0.85
Difficulty in mobility (*versus:* no)	0.83	0.73–0.93
Frequency with which one meets a friend *(versus*: one doesn’t have friends or meets them less than once a year)		
1 or 2 times a year	1.08	0.77–1.51
At least once, every 2–3 months	1.25	1.03–1.52
1 or more times per week	1.36	1.17–1.58
Instrumental support indoors (*versus:* no one)		
Others	1.48	0.97–2.23
Other relatives	1.36	0.85–2.17
Children/ Daughter-in-law or son-in-law	1.51	0.99–2.31
Spouse or partner	1.69	1.10–2.59
Emotional support (*versus:* no one)		
Others	1.22	0.92–1.62
Other relatives	1.45	1.13–1.85
Children/ Daughter-in-law or son-in-law	1.41	1.10–1.80
Spouse or partner	1.33	1.03–1.71

* Estimated by means of Poisson regression and adjusted for all study variables.

## DISCUSSION

In this study, a worse PQOL was observed among the elderly with higher schooling and mobility difficulties. On the other hand, the best PQOL was found among those who met their friends more frequently; who had instrumental support from spouses and emotional support from other relatives, descendants of the first generation and others and spouses. These associations remained after mutual adjustments and other relevant characteristics. The lowest prevalence of the best PQOL among participants aged 80 years or older is reported in the literature1^1^ ^,^ ^2^ ^,^ ^9^. This can be attributed to the fact that they are more exposed to associated disabilities, chronic diseases, chronic pain and depression^1–5^, and thus to more threat to their sense of control and autonomy than people under 60 years of age.

The worst PQOL among those with higher schooling seems to be counterintuitive and contradicts current data found in the literature^4^ ^,^ ^5^. One possible explanation is the low socioeconomic status and low social capital of these individuals with only the satisfaction of the most basic human needs, which would prevent them from meeting higher needs. People with this background may judge their own income as insufficient and, at the same time, give a high score to their quality of life. This positive evaluation possibly works as a protective element in the face of the negative effects of their dissatisfaction with their own quality of life, while the low PQOL score can work as a stressor, undermining personal resilience resources. Another possibility of managing the situation would be to adopt a lifestyle that is incongruent with one’s income, which could lead to increased psychosocial stress^15^.

More than half of the elderly reported having some degree of mobility limitation. Reduced mobility is associated with negative health outcomes, such as sedentary lifestyle, obesity, physical disability, poor quality of life and premature mortality^27^; being therefore a critical element in the well-being of the elderly. The association between absence of mobility difficulty and the best PQOL observed in this analysis is therefore consistent with the literature.

According to the theory of social-emotional selectivity, the network of social relationships decreases in old age^8^. Far from reflecting any social imperative of detrimental or unwanted disengagement or withdrawal for the elderly, this reduction is adaptive. In view of the diminishing prospects of future time, the elderly tend to maintain relationships that bring them emotional comfort and discard those that do not fit this description, usually the most peripheral ones. The same theory helps to explain why contacting friends is more important for the well-being of older adults and the elderly than contact with family members. While the former are free choice relationships, and can be selected based on affinities, in addition to not being stressful, the second ones are mandatory and may impose unpleasant situations of social interaction that are intrusive, exhausting and hardly preventable^8–12^. Activities performed with the family cause an increase in positive and negative affect, but do not increase satisfaction, while those performed with friends increase the positive affects and decrease the negative ones, leading to an increase in satisfaction^7^.

The acceptability of social supports is a complex issue, influenced by age, sex, health conditions and the functional capacity of care recipients, by the current stereotypes about aging, social expectations and the willingness of network members to offer support. In general, older people tend to accept more emotional support from friends than from children and other relatives, and tend to accept instrumental and material support from the spouse and children more than that from outside the family^7^. Confirming the important place of the family as a relational center and locus of social support for the oldest individuals, this study revealed a higher prevalence rate of high PQOL among participants who expected to receive material support from descendants of the first generation, possibly for validating their social expectations of respect and reward and for strengthening their sense of self-actualization as parents. To receive instrumental and emotional support from any sources has been proven to be more important for the determination of PQOL than not receiving it from anyone. However, the highest prevalence ratios associated with receiving instrumental support from spouses and close relatives confirm the importance given to the family as a source of such support. Despite contradictions and family conflicts, accepting help from family members seems to be better for the well-being of the elderly than exposing their own dependency to people outside the family.

Elderly dissatisfied with the support received from the family have a great chance to live with depression, negative affects and feelings of loneliness^15^. If viewed as something undesirable and permanent, motivated by social isolation and lack of expected support, loneliness tends to be related to increased morbidity, mortality, and low PQOL^9,11–13^. If focused, however, as an affective experience modifiable by emotional support, as a product of the process of social-emotional selectivity or as a necessary experience for personal growth, loneliness can have a positive effect. In this study, the feeling of loneliness did not show a statistically significant association with PQOL.

There was no independent association between PQOL among older adults and the different levels of social participation. This contradicts expectations generated by data showing participation in more complex social activities as an element that favors the evaluations of PQOL, due to the association with greater independence and autonomy, better mental health, lower risk of loneliness^12^ ^,^ ^13^, and more refined interpersonal skills and problem-solving abilities^27^. Thinking that the involvement in AADL (Advanced Activities of Daily Living) is also a question of motivation and social-emotional selectivity and that engagement in more complex social activities depends on one’s educational level and social capital, may help to interpret apparently incongruent data.

Studies that focused on the relationship between socioeconomic position and PQOL in people aged 60 years and over reported a strong association between low socioeconomic status and low PQOL. However, these associations appear to be weak when adjusted for social support. That is, even if impaired by low objective or subjective health condition, poverty and lack of opportunities, the elderly can enjoy the protective benefits of informal social support regarding the overall view of adjustment itself and of life as a whole^28^ ^,^ ^29^. To have larger friends’ networks, to have a partner, to have a confidant, and not to negatively rate one’s closest relationships increase the PQOL28^28^. The size of social network and frequency of contacts with the network are positively related to PQOL, after controlling for confounding factors such as socio-demographic characteristics, socioeconomic factors and chronic diseases ^18^.

In future studies, it will be interesting to analyze the performance of each of the CASP-19 factors in relation to the psychosocial variables considered in the present investigation. The inclusion of chronic diseases and associated physical disabilities, as well as variables such as ethnicity and housing arrangements, may help to explain the variations in PQOL and their factors, according to the variables age and sex, schooling and income. Finally, the use of advanced multivariate analyses may better enable the discrimination of the associations between the variables and the proposition of more satisfactory theoretical explanations for the data.

The ongoing increase in the longevity of the population observed in Brazil will require researchers to generate more knowledge to reconcile the conditions and concepts of good quality of life, frailty and care^30^. Multidimensional conceptions of health and well-being, with a place reserved for psychological well-being as a protective variable and as a variable that promotes development, will be a good starting point for new policies and social practices that are tuned to these new needs. Our results showed that, beyond age and schooling, mobility, sociability, and instrumental and emotional support are associated with PQOL among older adults. These characteristics must be considered in actions aimed at promoting the quality of life of this population.

## Footnotes

a Fundação Oswaldo Cruz. Brazilian Longitudinal Study of Aging. Rio de Janeiro; c2015 [cited 2017 Nov 28]. Available from: http://elsi.cpqrr.fiocruz.br
